# Supplementary material for: Spatiotemporal analysis of autism gene enrichment implicates cortex, thalamus, and hypothalamus
Source: bioRxiv. 2026 May 14:2026.05.14.724487. Preprint. [Version 1] doi: 10.64898/2026.05.14.724487 (PMC13192649; doi:10.64898/2026.05.14.724487)
Supplement: Supplement 1 [file media-1.pdf]

Supplemental Figures

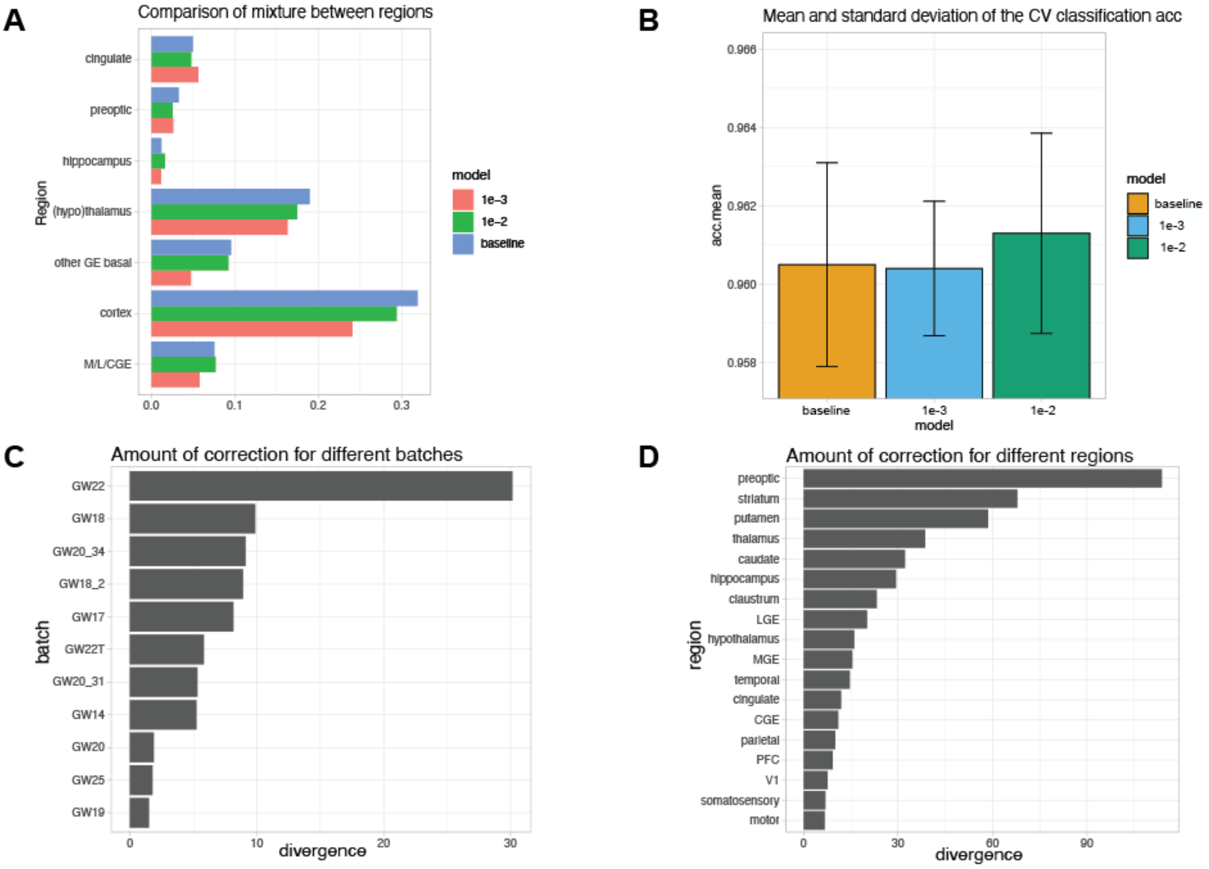

Supplemental Figure S1: Parameter selection and correction of sample and regional effects. **A)** Mixing scores for each area with model regularization $\lambda = 0, 10^{-2}$ and 10^{-3}; smaller lambda (or less regularization on the regional perturbation) does remove the regional correlations in the membership matrices, building the basis of downstream reasoning. **B)** Mean and the standard deviation of the CV classification ACC. **C)** Amount of correction for different batches, $\lambda = 10^{-3}$. **D)** Amount of correction for different regions, $\lambda = 10^{-3}$.

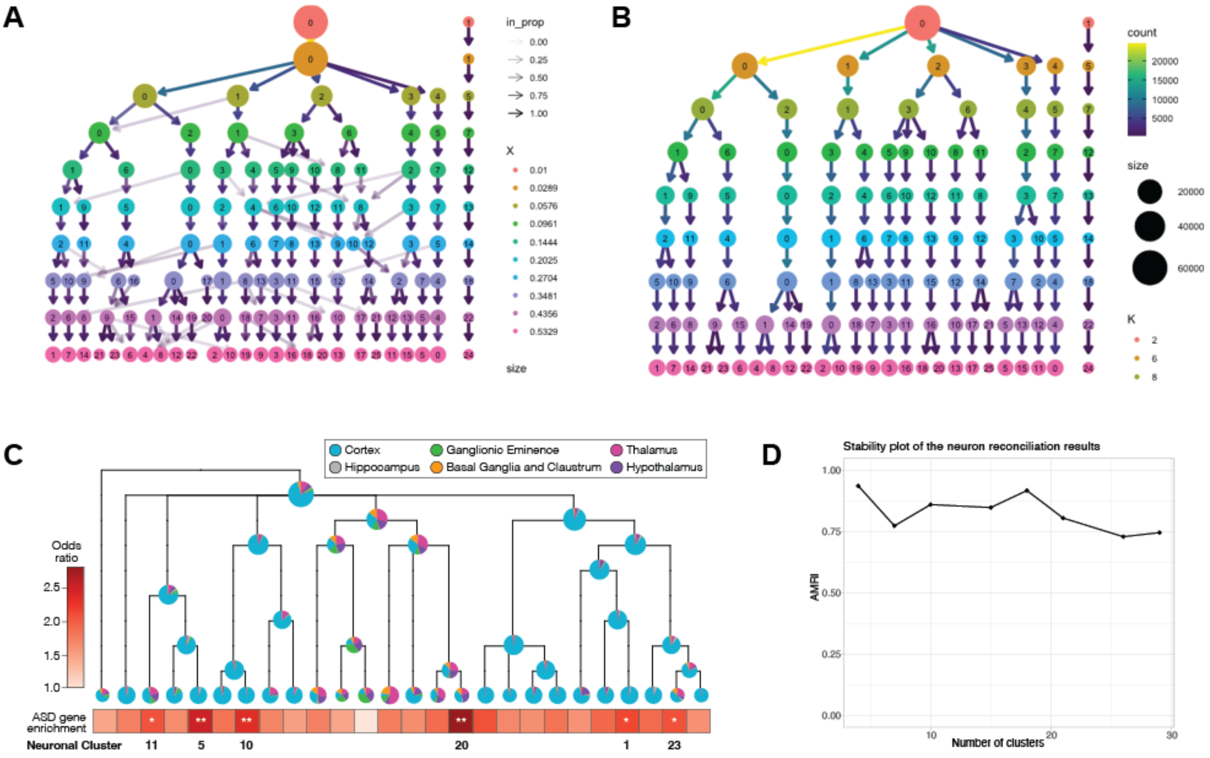

Supplemental Figure S2. MRTree clustering of excitatory neurons. **A)** The clustering results from FindMarker() (Seurat v5.0.3), estimated separately for each layer. **B)** The reconstructed hierarchical tree using MRTree. **C)** Regional composition of the nodes on each level along with ASD risk gene enrichment of the 26 leaf nodes. **D)** Stability plot of the number of clusters, indicating that the clustering results are robust to the parameter choices. The leaf level of the MRTree produces 26 clusters for the excitatory neurons, which was used for subsequent analyses.

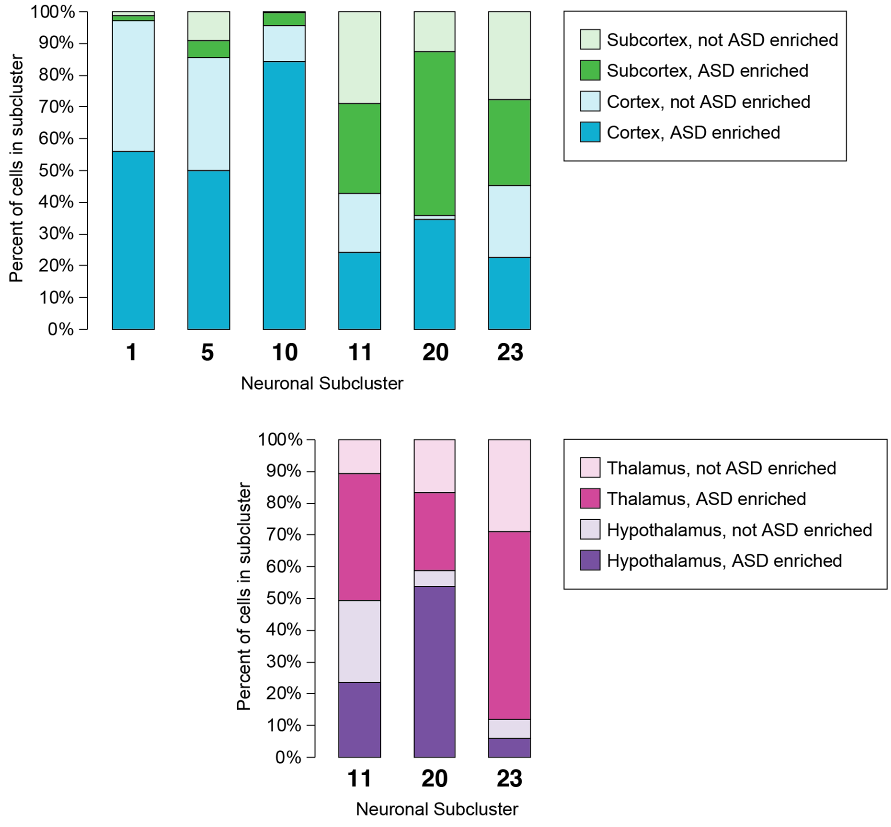

Supplemental Figure S3. Enrichment of ASD-associated genes. Excitatory neurons in the cortex (blue) and subcortex (green) are colored by the presence (darker shades) or absence (lighter shades) of enrichment for ASD-associated genes. Similarly, excitatory neurons in the thalamus (pink) and hypothalamus (purple) are colored by the presence (darker shades) or absence (lighter shades) of enrichment for ASD-associated genes.

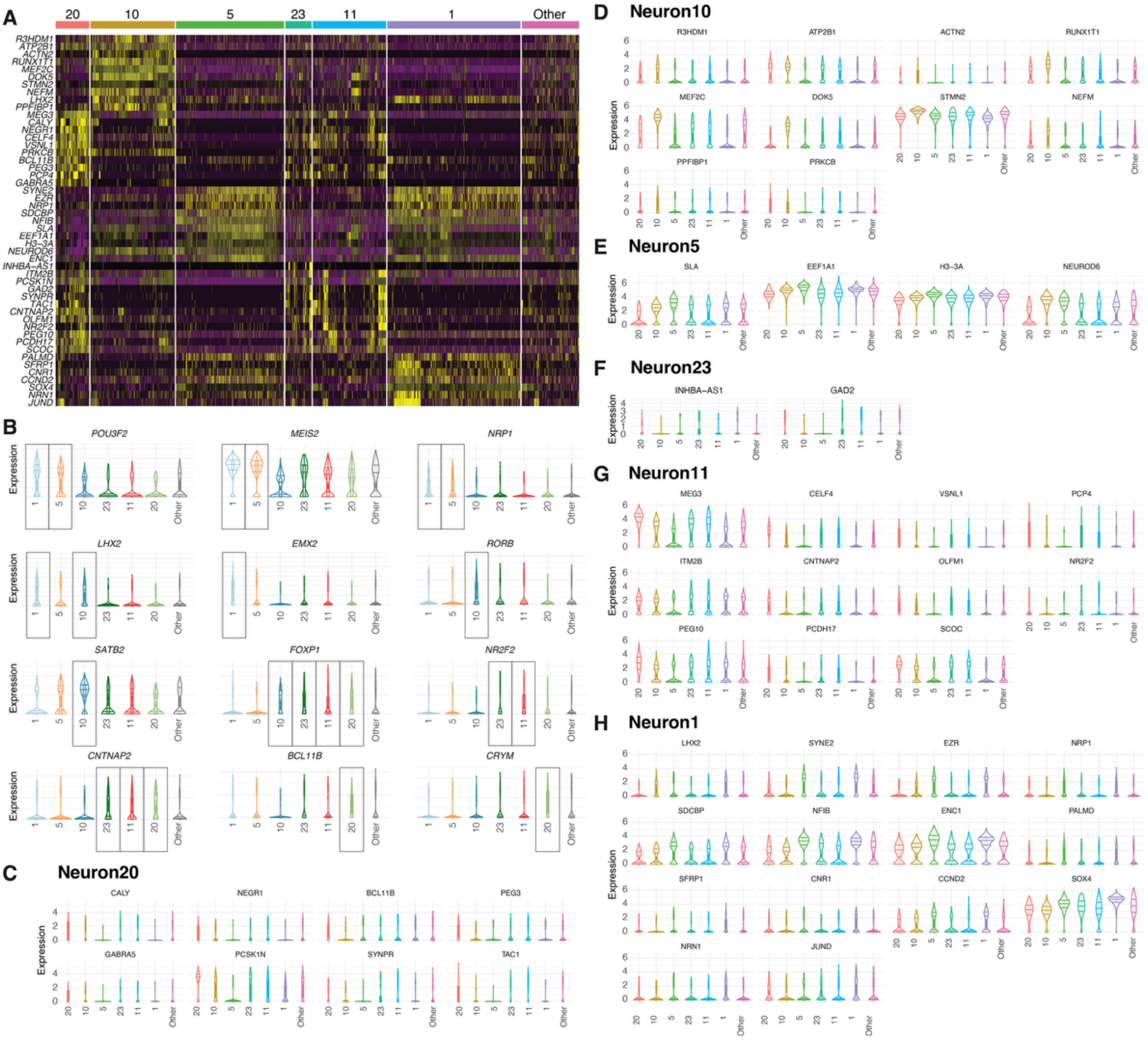

Supplementary Figure S4. Marker genes for ASD-enriched neuron subtypes. A. Heatmap of subtype specific marker gene expression for ASD-enriched neuron subtypes. **B**. Violin plot of known cell type markers and their expression in the ASD-enriched neuron subtypes. **C-H.** Violin plot of subtype specific marker gene expressions for the ASD-enriched neuron subtypes.

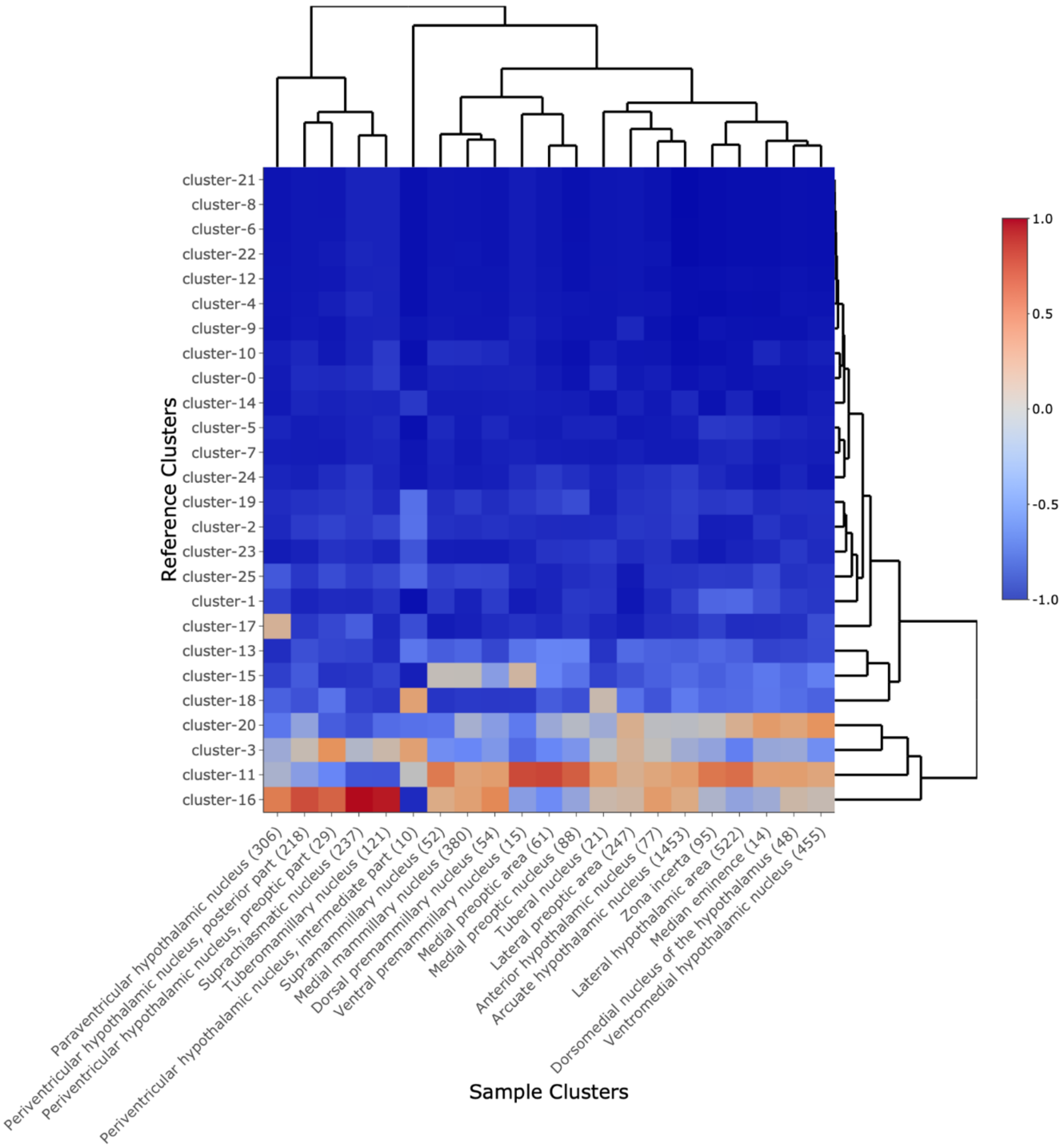

1,2

Supplemental Figure S5. Mapping of subclusters to hypothalamic clusters from HypoMap. Developing human excitatory neuronal subclusters were projected onto hypothalamic clusters in HypoMap, with prediction scores for cluster matches shown as a heatmap, scaled by hypothalamic cluster. Subcluster 20, which showed high enrichment for ASD-associated genes and a large proportion of neurons from the hypothalamus, matched strongly with several regions including the median eminence, dorsomedial nucleus of the hypothalamus, and ventromedial hypothalamic nucleus. Numbers of cells in each hypothalamic cluster are indicated in parentheses.

Major Regions	Regions
Cortex	PFC
	Motor
	Somatosensory
	Parietal
	Temporal
	V1
	Cingulate
Hypothalamus	Preoptic
	Hypothalamus
Basal Ganglia and Claustrum	Striatum
	Putamen
	Caudate
	Claustrum
Ganglionic Eminence	LGE
	CGE
	MGE
Thalamus	Thalamus
Hippocampus	Hippocampus

Supplemental Table 1. Major regions and corresponding regions in the Bhaduri dataset.

Gene	p-value	Avg_log2FC	Adj p-value	cluster	gene.name
**ENSG000000489912**	0.000000e+00	1.1413112	0.000000e+00	10	*R3HDM1*
**ENSG000000709612**	0.000000e+00	0.8624473	0.000000e+00	10	*ATP2B1*
**ENSG00000077522**	0.000000e+00	1.3305071	0.000000e+00	10	*ACTN2*
**ENSG000000791022**	0.000000e+00	1.5612273	0.000000e+00	10	*RUNX1T1*
**ENSG000000811893**	0.000000e+00	1.6275406	0.000000e+00	10	*MEF2C*
**ENSG000001011343**	0.000000e+00	1.6136567	0.000000e+00	10	*DOK5*
**ENSG000001044354**	0.000000e+00	0.6438235	0.000000e+00	10	*STMN2*
**ENSG000001047222**	0.000000e+00	1.3676967	0.000000e+00	10	*NEFM*
**ENSG00000106689**	2.027951e-290	1.1310808	6.923425e-287	1	*LHX2*
**ENSG000001108411**	0.000000e+00	1.4003115	0.000000e+00	10	*PPFIBP1*
**ENSG000002145482**	3.230490e-109	0.9195507	1.102889e-105	11	*MEG3*
**ENSG000001306431**	1.085761e-296	2.1174005	3.706786e-293	20	*CALY*
**ENSG000001722602**	1.435167e-285	1.4875034	4.899660e-282	20	*NEGR1*
**ENSG000001014892**	2.514454e-106	0.9071810	8.584347e-103	11	*CELF4*
**ENSG000001630321**	4.197787e-87	0.9390323	1.433125e-83	11	*VSNL1*
**ENSG00000166501**	1.474478e-232	0.9722231	5.033867e-229	10	*PRKCB*
**ENSG000001271523**	2.362602e-257	0.8032720	8.065924e-254	20	*BCL11B*
**ENSG000001983002**	4.402825e-255	1.6076137	1.503125e-251	20	*PEG3*
**ENSG000001830361**	4.997348e-85	0.7456092	1.706095e-81	11	*PCP4*
**ENSG00000186297**	4.564354e-250	1.2517788	1.558271e-246	20	*GABRA5*
**ENSG00000054654**	0.000000e+00	1.6285767	0.000000e+00	1	*SYNE2*
**ENSG00000092820**	0.000000e+00	1.3823014	0.000000e+00	1	*EZR*
**ENSG00000099250**	0.000000e+00	1.3671946	0.000000e+00	1	*NRP1*
**ENSG00000137575**	0.000000e+00	1.0601902	0.000000e+00	1	*SDCBP*
**ENSG000001478621**	0.000000e+00	1.0967165	0.000000e+00	1	*NFIB*
**ENSG00000155926**	0.000000e+00	1.3214578	0.000000e+00	5	*SLA*
**ENSG00000156508**	0.000000e+00	0.6551033	0.000000e+00	5	*EEF1A1*
**ENSG00000163041**	0.000000e+00	0.5559999	0.000000e+00	5	*H3-3A*
**ENSG000001646002**	0.000000e+00	0.7928197	0.000000e+00	5	*NEUROD6*
**ENSG00000171617**	0.000000e+00	1.0974793	0.000000e+00	1	*ENC1*
**ENSG00000224116**	4.439962e-185	1.4395486	1.515803e-181	23	*INHBA-AS1*
**ENSG00000136156**	3.066431e-153	0.9970797	1.046880e-149	11	*ITM2B*
**ENSG000001021096**	3.532679e-97	0.9080235	1.206057e-93	20	*PCSK1N*
**ENSG000001367501**	7.764232e-51	1.2086886	2.650709e-47	23	*GAD2*
**ENSG000001636302**	2.397409e-86	0.4174026	8.184753e-83	20	*SYNPR*
**ENSG000000061282**	1.368571e-101	1.6067870	4.672301e-98	20	*TAC1*
**ENSG000001744691**	9.766431e-82	0.9290922	3.334259e-78	11	*CNTNAP2*
**ENSG00000130558**	2.834704e-29	0.4641852	9.677680e-26	11	*OLFM1*
**ENSG00000185551**	5.305655e-304	2.1514963	1.811351e-300	11	*NR2F2*
**ENSG000002422651**	1.563009e-101	1.2027769	5.336111e-98	11	*PEG10*
**ENSG000001189461**	1.606920e-85	0.9130276	5.486026e-82	11	*PCDH17*
**ENSG000001531302**	1.717792e-84	0.6648287	5.864541e-81	11	*SCOC*
**ENSG00000099260**	0.000000e+00	1.2670068	0.000000e+00	1	*PALMD*
**ENSG00000104332**	0.000000e+00	1.4003975	0.000000e+00	1	*SFRP1*
**ENSG00000118432**	0.000000e+00	1.2278695	0.000000e+00	1	*CNR1*
**ENSG00000118971**	0.000000e+00	1.0545852	0.000000e+00	1	*CCND2*
**ENSG000001247661**	0.000000e+00	0.8178123	0.000000e+00	1	*SOX4*
**ENSG00000124785**	0.000000e+00	1.5987086	0.000000e+00	1	*NRN1*
**ENSG00000130522**	0.000000e+00	1.4097674	0.000000e+00	1	*JUND*

Supplementary Table 2. Marker genes of ASD enriched neuron subtypes.

Cluster	Odds Ratio	p-value	Adj p-value
**Neuron10**	2.187	0	0
**Neuron20**	2.808	0	0
**Neuron5**	2.405	0	0.002
**Neuron23**	1.822	0.001	0.023
**Neuron11**	1.801	0.002	0.035
**Neuron1**	1.914	0.002	0.035
Neuron12	1.836	0.006	0.107
Neuron18	1.560	0.008	0.145
Neuron7	1.605	0.021	0.353
Neuron2	1.492	0.029	0.462
Neuron6	1.576	0.033	0.493
Neuron8	1.490	0.055	0.769
Neuron15	1.229	0.396	1.000
Neuron3	1.384	0.177	1.000
Neuron19	1.313	0.195	1.000
Neuron13	1.343	0.129	1.000
Neuron16	1.216	0.326	1.000
Neuron14	1.144	0.514	1.000
Neuron0	1.363	0.144	1.000
Neuron4	1.196	0.378	1.000
Neuron9	1.034	0.836	1.000
Neuron22	1.281	0.233	1.000
Neuron21	1.174	0.407	1.000
Neuron17	0.347	0.000	0.000
Neuron24	0.970	1.000	1.000
Neuron25	0.966	1.000	1.000

Supplementary Table 3. Enrichment of excitatory neuron subtypes, including odds ratios, p-values, and adjusted p-values. The top six excitatory neuron subtypes have an odds ratio greater than 1 and an adjusted p-value less than 0.05.
